# Book Reviews

**Published:** 1817-07

**Authors:** 


### CRITICAL ANALYSIS
### *OF RECENT PUBLICATIONS,*
### IN THE
### DIFFERENT BRANCHES OF PHYSIC, SURGERY, AND MEDICAL PHILOSOPHY.

---

*Practical Observations in Surgery and Morbid Anatomy; illustrated by Cases, with Dissections and Engravings.* By JOHN HOWSHIP, Member of the Royal College of Surgeons in London, and of the Medico-Chirurgical Society. 8vo.—Longman and Co. 1816.—pp. 483.

IN a short introduction the plan of this work is explained, which consists of a series of cases, interesting, well related, and all of them concluded with practical remarks. To do justice to the writer, we shall select a portion from each division, in the order in which we find them. The following, among other interesting brain cases, may serve to show the importance importance of attending to the slightest symptoms of disease, particularly in those organs, without the regular operation of which, life is neither desirable nor can be long preserved.

"*Slight Injury to the Head, producing Symptoms, and ending fatally near Forty Years afterward.*

"In 1792, I was desired to examine the head of Mrs. E———n, who had died the day before, and whom I had attended with Dr. Turton and Dr. Harvey, about eight months previous to her death, having made her various setons, issues, &c. by their direction. Her case and the appearances were the following.

"She was about fifty; the widow of the late Bishop of D———. When about fifteen, being at play, she received a slight tap, rather than a blow, on the right side of the head. It gave her at the moment rather severe pain; but she disregarded it, and no immediate consequences of any kind followed more than a common headache, commencing always in the part stricken.

"For above thirty years after, she was subject to these attacks, and then began to grow heavy, and sometimes stupid and sleepy, without any known additional cause, though she was naturally one of the liveliest, and most witty women existing.

"This disposition continued gradually increasing till, for the last year and a half, it was very difficult to keep her awake; but, when she was awake, as I have often known, though it was but for half an hour, she had all her natural brilliancy of conversation about her; then all at once would drop asleep again, not to be roused. In this way, she went on till a perpetual comatose state took place, and she died convulsed.

"Latterly her vision had become very much, although very gradually, impaired.

"*Examination.*—On examining the head, as soon as I had removed the scalp over the right parietal bone, I saw a portion of the bone, about the size of a crown-piece, seemingly of a very dark colour, directly under the part where the blow had been originally received, and to which spot she invariably pointed as the seat of her pain. On removing the right parietal bone, I found that part of it which appeared discoloured was transparent, and almost wholly absorbed. It had that colour given it from the portion of the right hemisphere of the brain directly under it being perfectly black, and the colour appearing through the bone, for the dura mater at this part was altogether removed by absorption. Had she lived much longer, I am clear the bone also would have been altogether absorbed, and the brain itself protruded.

"The portion of brain under the seat of the injury was indurated, and scirrhous, and this change had taken place through the whole of the middle lobe cerebri. The colour was a dark livid hue.

"Every other part of the cerebrum and cerebellum were perfectly sound, nor was there any disease whatever in the contents of the thorax, or abdomen. Nothing but the disease above described, which had so pressed on the optic nerves at their origin as to have made them as flat as a piece of tape, and thereby occasioned her loss of sight, which amounted to almost total darkness for some time before she died.

"How far the tap of the cane, almost forty years before, brought on this train of symptoms, it may not be easy to decide, and yet it should seem to have had a part in it, by the pain having never varied from the spot where the blow was originally given.

"*Slight Injury to the Head, producing Internal Mischief, and ending fatally Six Years afterward.*

"A young gentleman, at twelve years of age, received a rap at school with the edge of a flat ruler, because he was dull at his learning. The blow was on the right side of the head, and a small wound was the consequence, which, for the space of six years, nothing would heal. It then healed, and he very soon afterward perceived that his sight was beginning to fail. In this respect he continued to decline, till at length he became quite blind. Added to this complaint, he now began to suffer from epileptic fits, which most frequently returned upon him every day.

"In the above unfortunate state he was brought up to London to consult Dr. Lettsom and Mr. Heaviside. On examination, there was no particular appearance found in the cicatrix of the old wound, where the blow had been received.

"The only thing that was considered likely to afford any prospect of real advantage, was the removal of a portion of bone by the trephine, to come at once, if possible, at the seat of the mischief. This was determined upon, and the operation performed.

"On exposure, the bone was not found diseased, nor even discoloured. On removal of the piece separated by the crown of the trephine, some blood and serous fluid escaped from between the skull and dura mater. This membrane, however, did not appear to have lost its healthy colour. By the next day the pupil of each eye had recovered its natural sensibility, dilating and contracting, according to the degree of light. The blindness unfortunately remained absolute, as before the operation.

"No favorable change took place from what had been done for his relief, but on the contrary his strength hourly decreased, a degree of low fever supervened, and on the third day after the application of the trephine, he was seized with an unusually severe fit, and very soon afterward expired.

"*Examination.*—On opening the head, the cranium was to appearance every where healthy, and so was the dura mater. Below the part where the dura mater had been exposed by the trephine, and consequently opposite the seat of the original wound, the pia mater had evidently suffered from chronic inflammation, but this appearance was circumscribed.

"On cutting into the brain, it was found indurated to a considerable derable degree, and this induration had extended itself, as in the last mentioned case, to the whole of the middle lobe of the cerebrum. It commenced upon the surface of the hemisphere, passing through the brain, down to the basis of the cranium.

"There were no other morbid appearances.

"The two last cases are calculated to convey a useful lesson to young practitioners; they show how cautious we should be in venturing an absolute opinion as to the certainty of a person having nothing to apprehend in the future from injury to the head. In the first case particularly, it is extremely curious that a disposition to mischief should have remained, as it were, suspended over a part for so many years together, as it seems scarcely warrantable to suppose disease could have actually been going on, during the long period of years of total exemption from pain and inconvenience, which this lady enjoyed."

Of the next chapter, *On some Diseases of the Neck*, affections of the larynx occupy the first place; and, as we have had several occasions to notice croup in some of our late Numbers, we transcribe the following in the author's own words.

"An inflammation of the mucous membrane lining the cavity of the trachea has been called croup, and, from a certain peculiarity in the sound of the voice being considered a constant attendant, this symptom has been very generally pointed out, as the diagnostic sign of the disease. The inconstancy of symptoms, however, is such that it may be almost doubted whether there really are any symptoms that can be absolutely relied upon to point out the existence of particular internal disease. I have seen a case of croup, in which this symptom was wanting, and on this very account, the true nature of the affection was not suspected till it was too late. I have attended in another, where, from the presence of this symptom, the medical gentleman that was first in attendance was clear the complaint must be croup, and the disorder, which was not croup, was consequently aggravated by the means used for its relief.

"*Inflammation of the Trachea.*

"The former of these two cases was in a child about eight years old, who had been attended by a physician of high reputation and much discernment. There were the general symptoms of fever, quick pulse, head-ache, &c. with some oppression, but no pain about the chest. On the turn of the first week, some degree of redness and inflammation took place about the fauces, the tonsils became sloughy, and the tongue lost its former white colour, and became first brown, and then black. At the end of the fortnight, the child was rapidly declining. Respiration then became extremely laborious, and this continued little more than twenty-four hours before the child expired apparently suffocated.

"At the commencement, the common medicines for fever were

 administered, administered, and, when latterly it became an object to raise the strength of the constitution, wine, bark, and opium, were prescribed.

" *Examination.*—I was requested to be present at the opening of the body, on which occasion the fauces and tonsils were found inflamed. A small slough had nearly separated from the right tonsil; but the opening into the larynx seemed also inflamed, and almost choaked up with mucus. The larynx and trachea were, therefore, removed and laid open, when the whole extent of the membranous lining was found highly inflamed.

" Upon the internal surface of the membrane lining the trachea, and very loosely connected with it, was deposited a considerable quantity of coagulable lymph. This was of a yellow colour, and pulpy consistence, which circumstances might have in some degree depended on its being mingled with a profuse secretion of viscid mucous matter. The coagulable lymph formed an irregular tube within the trachea, while the mucous matter, partly of a purulent colour, and partly clear and transparent, occupied the space within the adventitious lining. The mucous membrane itself, when exposed, was found of the brightest scarlet colour, from high inflammation.

" These appearances extended themselves very far down beyond the bifurcation of the trachea; and even in the smaller branches which the purulent action seemed scarcely to have reached, there was an unusually abundant secretion of mucus, the consistence of which was so viscid and tenacious, that it must have materially hastened the fatal termination of the disease, by preventing the passage of the air, and eventually producing suffocation.

" *Spasmodic Affection of the Larynx, supposed to be Croup.*

" In the year 1812, I was desired to see Master H., a fine child about four years of age. Almost a twelvemonth had passed since the commencement of his complaint. The symptoms had been, extreme difficulty in breathing, with a sort of ringing or stridulous sound in the throat. The medical attendant had at first pronounced it croup, and the usual remedies for that complaint, with blistering, leeching, &c. had been adopted in vain; indeed, the complaint seemed rather worse than better after them. In the course of a few days, the urgency of the first attack began to abate, and the child soon got the better of it. There had been no well-marked feverish paroxysm throughout, so that the circumstances of most importance were the fulness about the vessels of the head and face, arising from the occasional temporary aggravation of the dyspnœa.

" This little boy, when he had recovered, did not remain very long well; but the return of his disorder proved less severe, and less tedious than it was at first. The presence of the croupy sound in breathing, and the manifest obstruction to the transmission of air through the larynx and trachea, induced the practitioner still to maintain his first opinion of the disease.

" In

"In this way the child went on, sometimes better and sometimes worse, but occasionally altogether free from his complaint for a month tog ther; until accidentally the father, mentioning, in company, the child having had the croup for so long a time, was told it was no such thing; for that the croup was an acute disease, and always ran its course very rapidly. Soon after this, under the idea that the complaint had been misunderstood, I was requested to see the child; whom I found in every general respect healthy. There was, however, still some difficulty in breathing, dependent evidently on an obstruction about the larynx. The complaint was much better in clear and dry, than in close and moist weather; and, on inquiry, I found that affections of mind, particularly anger and passion, never failed to aggravate it most materially; and, that if the child was contradicted, or corrected, the dyspnœa became invariably so much worse, that he would quickly become black in the face, and frequently remained in that state for an hour, or more.

"Under the impression that the affection was purely spasmodic, I prescribed a mixture with bark, opium, and æther, in a light form, to be regularly given. By this plan, the complaint, in a few days, became evidently better, and in the course of a fortnight was completely and permanently removed."

We have some suspicion, that the first of these cases was the effect of cynanche maligna; at least, we have seen all these symptoms in a subject where the rest of the family had scarlatina. Dr. Heberden, in his Commentaries, has a similar remark.

As to the second, we confess ourselves not easily satisfied with the hackneyed expression of spasm or spasmodic affection, we should rather impute the symptoms to a slighter inflammation, which had become chronic, or was readily excited from very slight causes. Under such circumstances, inflammation is kept up by debility, and the remedies prescribed were very likely to relieve it, as we often find in chronic ophthalmia. Two cases follow of croup, or inflammation in the trachea in grown people: of cases similar to these, we have lately said so much, that, we shall detain our readers only to express our satisfaction at the following remark of Mr. Howship.

"From observation, I should say that, in all probability, spasm of the muscles of the glottis, is much less frequently concerned in the fatal event of these complaints, than was supposed by Dr. Cullen; although, with a candor worthy of so great a man, he admits, that he never succeeded in relieving the symptoms by the use of antispasmodic remedies."

Cases of abscess in these parts follow; such, also, have lately come under our consideration. Two cases of enlargement of the lymphatic glands of the neck, are related as the effect effect of mercury; the first suppurated, and was rather the consequence of an irritable habit, induced by mercury and an irregular mode of life. The author remarks, that the thyroid gland is rarely affected with acute inflammation. Two instances are, however, related; one of which advanced to suppuration and the patient recovered. Some affections of the external parts of the chest follow; among the rest, a very painful condition of the sternum is detailed with much interest. We should suspect the whole was inflammation seated in the cartilaginous part, as it is well known, that cartilage is a substance which will bear very considerable inflammation without suppuration. When the disease is seated in the bone, abscess is likely to follow; such is the second case referred to by Mr. Howship. We are surprised to meet with so few instances of inflammation of the heart, and still more at the author's scepticism relative to the frequency of the disease. That palpitations often occur, and even pains described by the patient as seated in that organ, without any real disease, cannot be questioned; but, if we were to give our opinion, we should consider carditis and its consequences as more frequent than is generally suspected. Mr. Howship remarks, that he has dissected only three instances of malformation in the heart: these were, however, all of them somewhat similar, yet by no means common occurrences. In all three the aorta communicated with both the ventricles; this produced the usual consequences of irregular circulation—dyspnœa and purple skin. In two, the author remarks, that the heat of the body varied with the complexion of the skin. It is much to be regretted that this was unnoticed in the third subject, who lived to the age of sixteen years.

The remarks on the diseases in the pulmonary system are well digested and judiciously related; some of the cases are, as the author calls them, singular; but, as these could neither be suspected, nor relieved if known, we shall confine our extracts to such as are more frequent, more easily detected, and capable of relief.

"Inflammatory action in the lungs, as in other parts of the body, varies in intensity, and, as it becomes less acute, or more local, its characters change; and that which was originally a general affection of the lungs, may become new modelled, assuming the characters of a local affection only, perhaps without pain, or other constitutional sympathy.

"Dr. Cullen mentions an effusion of blood into the general cellular texture of the lungs, as one of the occasional consequences of acute inflammation. I have examined the lungs after death in many cases of inflammation, but have never found this appearance. In one one instance only I have seen it, and that was entirely unconnected with inflammation. It was in a child who died at about four years old, of hooping-cough. I had expected, from the oppression, and other previous symptoms, to have found the trachea stuffed with mucous matter; but it proved otherwise, for the passages of the trachea and bronchiæ were very free. The apparent cause of suffocation was an effusion of blood into the cellular texture of the lungs. The appearance was that of large maculæ or spots of a deep red colour, shining through the membrane covering the surface of every part of the lungs. When cut into, the extravasated blood ran freely out. It seemed to have operated by compressing the air cells, in this way preventing the blood from circulating freely through the lungs.

"I have once seen violent inflammation produce an immense effusion of serous fluid into the cellular texture of the lungs, which very quickly proved fatal, the following are the particulars of the case.

"*Serous Effusion into the lungs.*

"A robust man, and a very hard drinker, about thirty years of age, complained in January 1809, of having taken cold. His pulse was soft and natural, but he had some oppression and an occasional pain in the chest. A blister was ordered, and some medicines.

"The following day, he said the pain was much better, and that his respiration was very free. He was, therefore, directed to continue the medicines that had relieved him, and put his feet into warm water at night. During the evening, however, his breathing became suddenly worse, and his complaint now rapidly increased. He said he had no pain, but an extreme oppression, and sense of suffocation about the breast.

"At a late hour in the evening, he had entirely lost the power of speaking. The pulse was soft, small, and regular, at about 90. There was a cold clammy moisture upon the skin. The countenance was full of despondency. The respiration was noisy, and laborious beyond description.

"The following morning he was still alive, and had coughed and spit up some thin frothy matter. From the suddenness of his attack the preceding night, it was considered not improbable that a collection of matter had burst into the lungs, and, in this view, his living through the night was more than was expected. The difficulty of breathing continued to be extreme the whole of the day, but towards the evening he was still worse, and the countenance was manifestly changing. The pulse now became extremely low and weak. He fell into a comatose state, and expired before the morning.

"*Examination.*—On opening the chest, adhesions were found in various parts, apparently of remote date. The structure of the lungs, however, was sound; and readily yielded to pressure. On cutting into the lungs, an astonishing quantity of thin, frothy, mucous cous fluid poured out; this seemed to flow principally from the air cells and cellular texture of the lungs, for, when the lungs were squeezed, it was seen to issue thence clearly. In some parts of the lungs, the same fluid was found in the bronchial ramifications, and was evidently the same with the matter expectorated. Wherever the substance of the lungs was cut into, that part of the section nearest the surface immediately poured out copious streams of fluid matter, apparently of a mediate consistence, between pus, mucus, and serum. It was extremely thin, and limpid; it was frothy, yet flowed out like water, although here and there it was tinged with a streak of faint yellow, as if from the admixture of purulent matter."

We have copied this passage, that the reader may compare the first paragraph with the last. Inflammation of the substance of the lungs rarely induces any considerable pain, as the author remarks; but, if it continues long, it becomes much more dangerous. Instead of adhesions, as in inflammation of the pleura, in which the pain induced never fails to give alarm, we have effusions into the lungs which suffocate before the patient or the inexperienced practitioner is sensible of the danger. Some remarks follow on hæmorrhage from the lungs, in which a case is described of such a discharge being vicarious for the menstrual, from the forty-fourth to the forty-sixth year of the patient's age. This is less remarkable at that age, as before and during such changes many anomalous symptoms occur, which, in our opinion, are too much under-rated. The following case is, in many respects, similar to one related in the Philosophical Transactions.

"James Butler, aged sixty-five, employed by Chippendale, of St. Martin's-lane, was working in the repair of an ornamented ceiling. He had two nails in his mouth, and, while looking upward at his work, a little irritation set him coughing, when one of the nails was thrown out of his mouth, and the other in recovering his breath, to use his own words, 'slipt down his wind-pipe.'

"Incessant irritation, pain, and cough, directly followed, and so continued till the man was worn away to a skeleton, spitting up blood and mucous phlegm.

"All the faculty who were consulted pronounced his case hopeless; and, if rightly represented, (in themselves convinced, that had such an occurrence taken place, it must quickly have proved fatal,) assured him it went down into the stomach, and must have passed off through the bowels. They said that what he experienced arose from the irritation it produced when in the stomach, but that it was not in his lungs, as he imagined, or suspected.

"Dr. Pitcairn, Mr. Cruickshanks, and others, saw him. Mr. K. of St. Martin's-lane, was with him directly after it happened. Prescriptions mitigated his sufferings a little, but could not remove them. The pain and all his complaints were fixed in the right lobe of the lungs, and he could then, as at the first instant after the accident, cover the exact spot with his hand. Spitting of blood continued to recur at intervals, and the poor fellow was consigned to certain death.

"This lasted from the 15th of April, to the 12th of August following, when, after a copious spitting of blood, with a sudden fit of coughing, he threw up something with violence towards his teeth, against the roof of his mouth, mixed with blood. Perceiving it a hard substance, and ever having the nail in his mind, he spit into his hand, and found it to be the identical nail that had slipt down the trachea so long before. The head of the nail was rusty when thrown up.

"April 1815. Eleven or twelve years have clapsed since this event took place, and the man has enjoyed pretty good health; subject, however, to occasional cough, slight spitting of blood, and a painful sensation precisely in the old spot."

In his remarks on diseases of the abdominal cavity, we conceive the author runs too much into a common opinion concerning the mysenteric glands. In Case 67, p. 224, we are more at a loss than ever to find the slightest connection between the supposed cause and the effect, or even exactly to ascertain either. It would give us much pleasure to be favored with a communication on this subject, for which we are the more desirous as the history throughout is involved in an obscurity very different from the general tenor of the work. This chapter contains some remarks, ingenious if not new, on the effects of peritoneal inflammation. The complaints of the liver are such as have been often described —induration, abscess, and hydatids. Among the diseases of the intestinal canal, a case occurs in which a living worm escaped through an aperture in the parietes of the abdomen during life. This history has some other peculiarities well worth recording. A case of hernia is described, which the author speaks of as very rare. We have, however, seen two such, in which the intestine is strangulated in the openings of coagulated lymph, the effect of previous inflammations. One follows still more rare, excepting in very young subjects, in which the intestine was strangulated in a preternatural opening of the mysentery. Cases of hæmorrhage from the intestine follow, and one of a peculiar secretion voided by the rectum. This was no other than the adipocere which Sir Everard Home so lately offered to the public in his paper on the formation of fat. This part of the work concludes with some useful practical remarks, illustrated by cases of the diseases in the rectum.

The succeeding chapter commences with the "Passage of the Testicle into the Scrotum." On this curious subject we were prepared to expect some useful information, but the only cases related are, of the late descent of one of the testes. In all these subjects, the parts were left entire; that is, the patients recovered without an operation; so that, whatever obscurity might attend them was never developed. The diseases described in the testicles and scrotum are not new. The author's "Account of some Diseases of the Uterus and its Appendages," commences with that very frequent and often dreary complaint, the ovarian dropsy. Here we are presented with something like a theory, the best part of which is its brevity, though the conclusion is somewhat abrupt. We could supply our readers in a moment with two or three other theories, if we had not profited by the example of the author and of a good many other writers. The last related case, in which the operation of tapping was performed, contains an incident which we think it right to notice.

"In withdrawing the canula, (says Mr. Howship) the slit in the extremity of the tube was found to have pinched the adjacent margin of a membranous expansion, a small part of which was unavoidably drawn out with the canula, before it could be perceived, and, when the end of the tube was disengaged, in the moment of turning round to lay down the canula, a considerable extent of this substance was protruded. The occurrence was new, and it was proper to conceal it from the patient, while, at the instant, a doubt passed through my mind, what should be done with it, provided it could not be returned. On unfolding, it appeared so moderately vascular, that I determined, if its removal should appear necessary, tocut it off with a pair of scissors, in the mean time endeavouring to pass a part of it back, and fortunately the attempt succeeded; for, although the opening bore a very small proportion to the size of the protruded mass, it was returned with the greatest facility. This incident was neither attended nor followed by the least sensation of pain or uneasiness to the patient.

"The quantity of this prolapsed membranous substance was equal to a small pear. It was at first suspected it might have been part of a large hydatid, but a near examination of its structure, its vascularity, and general appearance, argued its being a part of a thin ovarian cyst. An hydatid possesses a uniform and dull colour, and is entirely destitute of red vessels; but this substance had many small vessels ramifying upon it, having also the bright and variable hue which sometimes is observed in an ovarian cyst."

Let us now attend to the dissection.

"On laying open the cavity of the abdomen, the viscera in general were found healthy; neither was there the least trace of peritoneal inflammation. The intestines were in their usual situation, but there was no appearance of diseased ovarium. I, therefore, passed my hand down into the pelvis, and found a large cyst, which felt so thin and equal in texture as to exactly resemble a large large collapsed hydatid. When, however, this cyst was brought into view, it was found to be connected with the uterus.

"The parts were, therefore, dissected out, and the cyst turned out to be the right ovarium, which was dropsical, and, what is not very commonly observed, it had in this case formed a single cavity, capable of containing many pints of fluid. The external surface of the diseased ovarium was like the other expansions of the peritoneum, perfectly free from inflammation."

In considering a work, the object of which extends to almost the whole of the human body, a dissertation on hydatids would be out of place, we shall, therefore, take the case as we find it, and regret that advantage was not taken of the "drawing out so much of this membranous bag" as to continue the extraction as long as the substance, whatever it might be, was uniform, and "the patient remained free from the least sensation of pain or uneasiness." We will even go so far as to acknowledge we have sometimes felt a wish, that a very delicate pair of forceps should, at the end of such operations, be introduced through the canula, to seize, if possible, the interior surface of the posterior parts of such a cyst, whether it should prove hydatid or the ovarium. We would propose the attempt at drawing should be continued no longer than whilst it can be done without force, and without exciting the least sensation in the patient. That afterwards, if not detached by our very gentle attempts, it should be left out with no other provision than such as would prevent its return. We have even felt an inclination, when such tumors have appeared more solid, and we were satisfied they were attached to the thin parietes of the abdomen, that a small puncture should be made. If the contents are melicerous, this will be sufficient for the escape of them; if solid, the puncture may be enlarged, if we find, as is usual, that the cyst adheres to the peritoneum, and the latter to the external parts. What has been said can only be considered as a hint; it is, however, one we make no scruple to say it is our intention to pursue when a case offers favorable for the attempt, and which, without such an attempt, is considered by others as necessarily fatal. This part of the work concludes with an account of some diseases and malformations of the female organs. We were disappointed in meeting with nothing, in so judicious a writer, concerning the malignant ulcer, or, as it is usually called, cancer of the uterus. Probably, having nothing new to offer, he might wish to waive a mere description of unhappy cases, several of which must have occurred in his practice.

A chapter follows on lumbar abscesses, in which we think less credit is given to Mr. Abernethy's improved mode of treating those diseases than is due to that writer; some of Mr. H.'s cases are, however, very interesting and instructive. Cases of hip-disease follow. Among the complaints of the upper extremity, we have an interesting account of one which ended in anchylosis of the shoulder-joint: as our immediately preceding and present Numbers contain much about arteries, we transcribe the following account of

" *Spontaneous Cure of an Inguinal Aneurism.*

" Stephen Lewis, aged fifty, perceived a small swelling at the left groin in September 1811. His attention was first drawn to the part by a throbbing sensation in it, particularly when standing up, which sensation he compared to a 'beating like a heart.' It was then not larger than a pigeon's egg.

" It increased slowly till March 1812, when it had attained the size of a large orange. About this time the parts covering the tumor were attacked with erysipelatous inflammation; and, although this attack was, by proper treatment, soon relieved, he found the aneurismal tumor more frequently painful afterward than it had been before. Its pulsations were much aggravated in severity, and he also complained of more or less uneasiness and pain throughout the whole limb.

" His general health, however, being tolerably good, he was still able to move about the house, notwithstanding the swelling in the groin, continuing to increase, was, in September 1812, equal in size to a large melon, and was frequently productive of paroxysms of extreme pain and irritation.

" As the winter approached, these pains connected themselves with others of a spasmodic nature, shooting downwards through the muscles of the thigh and leg.

" In the early part of February 1813, he was upon the water-closet, and, in straining to pass a confined motion, he distinctly felt something give way within the tumor; and immediately afterward found that the scrotum, the upper part of the thigh, and the lower part of the abdomen, were swelling very extensively, while the projection of the original tumor apparently diminished in the same proportion.

" By the following day, the extent and quantity of the diffused swelling was enormous, and the pain, both in the aneurismal tumor, and in the surrounding parts, was almost intolerable; added to which, a considerable œdematous enlargement of the whole leg and thigh now served to increase his general distress.

" The extreme severity of pain and irritation was from day to day relieved by the frequent administration of opiates, the effects of which were occasionally assisted by fomentations to the parts.

" After some weeks' confinement to bed, the severity of his sufferings began to diminish, and by degrees he recovered so far as to be again capable of moving about the house.

" In November 1813, [at least eight months after the aneurism had had burst,] he took a violent cold and cough, with which, and an increased pain in the extended seat of the disease, he was confined to his bed for near three weeks, almost without sleep.

" During this attack he was seized with an unusually severe fit of coughing; when in a moment he felt something burst within the tumor, and, turning aside the bed-clothes, found the bed first, and immediately afterward the floor of the room, inundated with a discharge of excessively offensive, grumous, and putrid blood, which poured out from a part of the integuments that had given way.

" Mr. Heaviside, who had attended him regularly, visited him accidentally within a few minutes after the swelling had given way. The quantity of discharge was at least equal to several quarts, and it appeared very doubtful whether he could survive the first effects of so tremendous a crisis.

" For the space of three weeks after this event, the putrid contents of the aneurismal cavity continued to come away in such quantity, that it was found necessary to change his bed-linen every day. In the course of this period several new openings formed in the integuments, while the general parietes of the tumor became thinner and more flaccid from the frequent evacuation of large masses of grumous blood and lymph.

" By the end of the third week a quantity of sloughy membranous matter made its appearance at the largest opening, and this with care was by degrees separated and brought away, and proved to be a very large mass of thickened, sloughy, and putrid, cellular membrane.

" The removal of this substance was productive of so much irritation and exhaustion, that, notwithstanding every assistance, it was scarcely expected that he could live through the night; from that time, however, he began to mend.

" On the evacuation of the contents of this immense tumor, the principal object, of course, was to support his strength, so as to enable him, if possible, to bear up against so great a discharge of putrid matter. With this view warm jellies, soups, wine, &c. were exhibited in small quantities every half hour, with the most unremitting attention."

By these means the patient recovered, excepting that to December last the seat of the large opening has occasionally ulcerated superficially, the limb is also much weakened.

The remainder of the work contains remarks on diseases in the bones of the limbs and on dislocations. On these, we could dwell with much pleasure, would our limits permit, but we must now conclude with our most hearty thanks to Mr. Howship for the present he has made to surgery and to the public. If we have seemed to differ from the author, it will be understood that we have felt it our duty to select the only passages which we thought required our animadversion, which are few in so large a volume. It is much against our will, that we now feel obliged to express our regret gret at the number of well executed plates. In many instances we found the description quite sufficient, where the plates must increase the expense and consequently injure the circulation of so truly valuable a work.

---

*Some Practical Observations in Surgery: illustrated by Cases.* By A. Copland Hutchison, late principal Surgeon to the Royal Naval Hospital at Deal; Member of the Royal College of Surgeons in London, and of the Medico-Chirurgical Society; one of the Consulting Medical Officers to the General Penitentiary, Mill-bank, Westminster, &c. &c. 8vo. pp. 167. Callow, 1816.

This is one of the publications which have remained too long on our shelves, whether we consider the importance of the subjects, or the manner in which they are treated. To give a just notion of the whole, we shall consider each chapter in its order. That on amputation treats "Of the proper period for operating in gun-shot wounds." Here the author makes no scruple to recommend the immediate amputation of a limb which cannot be saved. The principal arguments are, that time for deliberation on the part of the patient only adds to his despondency; and that the mischief already done is likely to be more injurious than a well-conducted operation. Some hints are thrown out on the supposed superiority which the medical officers in the army are apt to assume over those of the navy. We hope they are ill-founded. One writer is often alluded to, whose work, by its date, should have preceded this. Copious as the arguments and remarks are, we will venture to suggest one which we do not recollect to have been sufficiently insisted on. It is urged that military men, particularly privates, are usually in the flower of youth, and in a condition of high animation, both which are causes to induce high inflammation. Is there not, then, too much care taken to prevent the loss of blood in some instances of operation after wounds which have been readily staunched? We have often remarked, when a surgeon has related a successful amputation, under circumstances particularly unfavourable, that, among the latter, has been the great loss of blood either before or during the operation. Has not that very loss of blood been among the causes of success? and, when it has not occurred, should not blood be taken away by means of the lancet?

The second consideration is "On the application of the tourniquet."

tourniquet." These remarks we shall give in the author's words.

"The proper distance for the application of the tourniquet, whether the part intended for operating on be situated above or beneath the knee or elbow, should, in my opinion, be nearly the same; namely, a hand's breadth below the groin or axilla. The next circumstance that claims our attention will be the kind of pad or cushion, to be applied underneath the strap or web of the instrument, for compressing the artery. I am in the habit of using a pad considerably smaller than is commonly employed by surgeons in general, being not thicker than a finger, and which I place somewhat obliquely over the artery, to preclude the possibility of its being displaced, by any direction that it may subsequently be found necessary to give the limb during the operation. It may be made by taking a few turns with a bandage about a rounded piece of deal of the circumference of a goose-quill, an inch and a half in length. After the pad is thus prepared, it should be stitched, to prevent any embarrassment from unrolling during its application; with about a yard of bandage left hanging from the compress, for the purpose of passing round the limb.

"Surgeons usually place the screw of the tourniquet immediately over the pad for compressing the artery; but I am of opinion that a more convenient situation for this part of the instrument, both as it regards the operator and assistant, will be on the outside of the limb, nearly opposite the course of the artery to be compressed; for, by this position, the pad will be less likely to be displaced by the web of the instrument, than when the screw part acts immediately upon it. I have seen gentlemen, who rank deservedly high in the profession, make use of a pad of a rolled-up five-yard bandage: at one period I was myself in the habit of using such large compresses, and might have continued in the use of them to this day, had not a very embarrassing circumstance occurred to me on one occasion.

A principal objection to the placing of a large pad over the artery is, that the web of the tourniquet passing over a surface so much elevated above the circumference of the thigh, a considerable angular space on each side of it will be left slightly, or not at all, compressed by the circular band of the tourniquet, however closely the instrument may be screwed. This will not only endanger the loss of a large portion of blood, from the divided ends of such vessels as may happen to traverse that space, a circumstance to be particularly guarded against in cases of debility; but the emission of blood must also embarrass, and obscure the view, of the young surgeon, in the subsequent parts of the operation."

In dividing the parts, Mr. H. proposes the largest possible quantity of flap; and that, before the retractors are removed, the bone should be scraped with a blunt scalpel, which he prefers to the knippers or file.

On securing the blood-vessels, the author is as minute in his his direction as the importance of the subject demands. We shall recapitulate them, and add our own remarks. The including the vein in the ligature is objected to, from the danger of overlooking the nerve. Some other cautions follow, showing the difficulty of discovering the nerve when the parts are bloody, and particularly if previous adhesions have taken place. On the mode of tying the ligature, much stress is laid that the noose should be at right angles with the artery. This we highly approve, and, in addition to the cogent reasons suggested by the author, shall add another, the inattention to which we strongly suspect once proved the cause of a secondary hæmorrhage. If the noose is not at right angles, the part of the ligature within the knot is longer than is necessary; and, should it by any means become at right angles, it may not be small enough to close the area of the artery. If arteries are wounded longitudinally, they should be cut transversely. When ossified, or if the operator discovers a calculous deposition, they require the needle, to include some surrounding parts.

"It is of essential importance (continues Mr. Hutchison) in amputation, that every artery from which there may be the slightest probability of an after-hæmorrhage should be tied, previously to bringing into final contact the wounded surfaces; for, should a small branch escape detection, it may, when warmth and re-action take place, bleed profusely, occasion the removal of the dressings, the re-opening of the stump, additional pain to the patient, and increase the general irritation, by the alarm usually resulting from unexpected hæmorrhage.

"With this view, therefore, it will be requisite that every coagulum of blood shall be carefully removed, as I have frequently discovered the mouths of very formidable vessels concealed by coagula, which stop the bleeding only till the parts become heated, and this substance is broken down by the constant impulse of the circulation. The sponge I have often found insufficient to remove these coagula from the orifices of such vessels, and I have been frequently compelled to employ the nails of the fore-finger and thumb to detach them from their adhesions."

The practice of washing the bleeding stump in cold water is objected to. One end of the ligature is advised to be cut off about an eighth of an inch from the noose, excepting those on the two principal arteries. These should be distinguished by knots at the extremities of each; and, if the noose is not detached at a proper time, the ulcerative process should be hastened by twisting the two ends together, so as to tighten the noose. The modern practice of cutting all the ligatures close to the noose is objected to; but the question is left undecided, excepting in cases of aneurism, where it is altogether reprobated.

As we have, in the present and in our late Numbers, been very much led to the consideration of securing arteries, we shall offer a few words on this part of the work. We are by no means satisfied of the necessity of taking up so many arteries, still less of the propriety of forcibly removing from a small artery a piece of coagulum so firmly fixed as to require the nails of the fore-finger and thumb for its separation. In military surgery we are aware of the necessity of greater security than in private practice. In the latter, the surgeon will remain long enough with the patient to meet any accident of that kind. We would also recommend, that, after the larger arteries are secured, the cut surface should remain some little time exposed before the sides of the flap are brought together. By these means, a greater stimulus for retraction and contraction appears to us imparted to the smaller arteries, and the blood over their extremities coagulates more firmly. There is no danger of preventing union by the first intent from this delay. Such a process is, in our opinion, rather retarded than accelerated by that very high inflammation which is likely to follow the immediate apposition of the cut parts. In many cases, too, we would rather encourage than prevent the loss of blood from the smaller vessels, should it continue to flow after the larger arteries are secured. The caution concerning twisting the ends of the ligatures appears to us unnecessary, as we have never found any inconvenience from their remaining even after the ulcerative process had separated the noose, and granulations had formed so as to detain the ligature when pulled. On all these occasions, we have left the parts to separate this extraneous body in their own way; and had no occasion to repent, however tardy the process may have seemed. On the proposed mode of cutting the ligature close to the noose, we have already given our opinion much at large. What we have now offered will be considered as a mere opinion, which the author cannot be offended at if it differs from his, especially as we admit the useful hints we have received from the rest of his directions.

The remarks "on forming the stump" are all very judicious. We particularly recommend them to the attention of the naval surgeon, but they are applicable to every kind of practice. Equally satisfied are we with the general directions concerning the subsequent "exposing and dressing the stump." But we conceive that from the time of the operation to the fifth or sixth day, when the dressings are to be first examined, some instructions are necessary to prevent too high inflammation in the parts brought into contact. Instead of the quantity of roller and woollen night cap at one time in use, and some of which is still retained, we would advise that the parts should be covered as slightly as possible, their temperature often examined ; and, when the latter is too great, they should be left uncovered by the bed-clothes ; for, while any thing more than the standard heat is excited, we repeat it, there is much more danger of too great than too little inflammation.

"The medical treatment after amputation" is unexceptionable. In this, as in all other cases, we can only attend to the symptoms as they occur, and direct our remedies accordingly. Some well-related cases follow, all of which afford useful instructions to the young practitioner, and hints by which we may all gain something. After this concession, we conceive the author cannot be offended if we refer the case of Farrol [see page 61] to those in which the loss of blood might have been greater, certainly without injury to the patient, who is described as a strong muscular man.

A case follows of "Amputation of the foot at the tarso-metatarsal articulation." The subject was a young African, whose feet were frost-bitten. The operation succeeded.

The case in which amputation was performed during the spreading of mortification, we consider so important, that we should gladly give it in the author's own words, if our limits would permit.

A chapter follows on a most important subject in military surgery, particularly in the navy, namely, the treatment of erysipelatous inflammation.

"During the period (says Mr. H.) that the surgical department of Deal Hospital had been under my superintendance, I find, on a reference to the hospital books, and my own private notes, that upwards of forty cases of erysipelas had been admitted;* and, with three or four exceptions, it has, according to my judgment, been that species of the disease styled by nosologists and systematic writers Erysipelas phlegmonodes."

"The epidermis or rete mucosum has been supposed by some writers to be the particular seat of erysipelas, whilst others confine its morbid action alone to the cellular substance; and a third class to both these parts. My own observation, however, has afforded me convincing proofs that, in the species of the disease now under consideration, its active and destructive influence will be found

"* I say 'the hospital books and *my own private notes*,' because the greater number of cases admitted had either other diseases marked against their names, or merely the very general one, *inflammation*, from some inattention in filling up the sick tickets which accompanied the men on shore."

more more especially directed to the reticular, or condensed cellular substance, forming the aponeurosis of the muscles, &c.

"In the genuine erysipelas phlegmonodes, pus is seldom formed in the substance of the adipose part of the tela cellulosa, exterior to the aponeurotic expansion, that is, between this membrane and the skin: its most common position is beneath these parts, and in immediate contact with the muscles. Those conversant with erysipelas phlegmonodes, must have frequently witnessed the destruction of the aponeurotic coverings as soon as suppuration or effusion had taken place, by discovering large portions of that membrane detached, and floating in these secretions when liberated by puncture or other means."

Here again we are under the necessity of expressing our objections to every premature attempt at an artificial arrangement of disease, usually called nosology. We are the more inclined to do so, because our author, with less than his accustomed candour, seems disposed to accuse his brethren, from whom he received these subjects, of inattention, in using only the general term inflammation. Setting aside the hurry with which these tickets are sometimes made out, we are ready, not only to acquit these gentlemen, but to approve of their caution; and we shall see that Mr. H. speaks with some hesitation as to nomenclature, but with none as to the indications for treating the disease as inflammatory.

"In conjunction (says he) with the usual medical treatment in such cases, topical blood-letting, by means of cupping, followed by fomentations, was the plan I pursued in the first few cases that came under my care;* and, in the very incipient stage of the disease, this may be all that is required to effect a cure by resolution; but, should the disorder advance to suppuration, insulation of the integuments, as has been before stated, will not unfrequently be the consequence."

In the more advanced stage of the disease, nothing was found so serviceable as longitudinal incisions down to the muscles, or wherever the pus or other fluid was lodged. For the more accurate detail of symptoms and treatment, we must refer to the work itself, selecting only the following passage, connected as it is with several most important questions.

"* This is not a new practice. Cupping and fomenting the affected parts in erysipelatous inflammation, is recommended by Wiseman, in his Chirurgical Treatise, vol. i. published in the middle of the 17th century.—Sydenham also recommends repeated bleedings and fomentations to the parts affected. See his Opera Universa, cap. vi. p. 279."

 "The

"The bark, so strongly recommended by Drs. Fordyce* and Wells,† and more recently by Sir Gilbert Blane,‡ I have never prescribed in this species of the disease, previous to the reduction of the attendant fever, and the abatement of inflammation by the above-stated remedies; but, in the only two cases of erysipelas erraticum § that came under my care, the early exhibition of the bark was attended by the most salutary effects. These two last-mentioned patients were, at their admission into the hospital, extremely emaciated, old, and worn out in the sea service."

When we find a remedy recommended by such respectable names, and, most of all by the author of "Diseases of Seamen," we cannot fail to treat it with due respect; and we can only account for the difference of modern practice by suspecting, as Sydenham so often reminds us, that some unknown property in the air alters the form of diseases, and renders a different mode of treatment necessary. It appears, by that sagacious philosopher, that such was the case even in the exanthemata, where the cause and nature of the disease could not be questioned.—We conceive that the condition of Mr. H.'s two last patients, under *erysipelas erraticum*, would have been sufficient to indicate the use of the bark without any reference to the name given by Dr. Bateman to a disease, one character of which is, that it usually "terminates favourably in a week or ten days."

The remaining subjects treated by Mr. H. are,—"History of a Popliteal Aneurism—The use of the Probe in any Operation in which a punctured Artery is tied—On Necrosis—Case of Abscess and Hydatids in the Liver—On Lumbar Abscess—On ununited Fractures. On all these we must be short, excepting the case of aneurism, "in which a new method of applying the ligature was practised,"—a subject so much connected with our late enquiries, that the reader will expect our opinion upon it.

Mr. H. begins with the mention of Dr. Jones's experiments, informing us, that, before Mr. Travers's "valuable papers published in the Medico-Chirurgical Transactions," his [Mr. H.'s] case had occurred, and was written; and that he had repeated Dr. Jones's experiments, with corresponding success, on the brachial arteries of two dogs, as far back

"* See the Transactions of a Society for the Improvement of Medical and Chirurgical Knowledge, vol. i. page 290.

"† See also vol. ii. of the same work, page 213 to 229.

"‡ Sir Gilbert Blane's excellent work on the Diseases of Seamen, page 600.

"§ See Dr. Bateman's Synopsis on Cutaneous Diseases, page 130."

as as the year 1800. Notwithstanding this, however, from what to us appears an ill-judged caution, the author was induced to adopt Mr. Travers's improvement of leaving the ligatures on the arteries for six hours, in order "to afford sufficient time for the first process of adhesion to be affected." The issue was, that hæmorrhage followed first from the inferior cut end of the artery, and afterward from the superior: the limb was taken off, and the patient died.

"The above statement goes to prove the important fact, as far at least as the authority of a single case can vouch,* that adhesion and obliteration do not follow the application of ligatures on the femoral artery of the human subject, when subsequently removed, and even after having been continued on for some few hours. How far this conclusion may be applicable to vessels of smaller dimensions, remains to be proved by those who may be inclined to put it to the test of experiment. It is a well-known fact, however, that the parietes of the smaller arteries are thicker and more dense, in proportion to their diameters, than those of larger dimensions. It is, therefore, reasonable to presume, that a stronger barrier will be opposed to the transmission of blood in the former than in the latter, after the consequences resulting from the constrictive powers of the ligature applied in the manner described; and hence the probability of the experiment being attended with greater success in the one than in the other."

This case is particularly interesting, and related with great candour. We therefore think it highly deserving our notice, and beg to solicit the very close attention of the reader.

Dr. Jones, in a passage to which we are referred, informs us, "that, in making experiments of this sort, the restoration of the circulation through the vessel should always be obtained, although, in a practical point of view, it may be an advantage for the internal parietes to adhere; and thus their wounded surfaces may escape that impediment to their adhesion, which a stream of blood passing between them must occasion in a greater or less degree." "I determined, however," says Mr. Hutchison, "not to trust to this *reasoning*." It is with great pain that we feel obliged to ask Mr. H. what he means by this *reasoning*? Is it not a matter of fact to which we are referred? and can it be said that "Dr. Jones's experiment has failed of success," because a supposed im-

"* Since this case occurred, the operation has been twice performed in one of the London hospitals; and, although the patients recovered, the operation was found objectionable on two accounts —first, that seventy hours were required for the obliteration of the artery—secondly, that the artery ulcerated where the ligature was applied, and secondary hæmorrhage ensued."

provement provement upon it has failed? We are ready to admit that Dr. Jones may have led to this error by assuming that the obliteration of the artery is the mere effect of the adhesive inflammation excited by the rupture of the internal coat. Still, however, the experiment should have been correctly conducted, and without any fancied improvements.

Let us remark, that the permeability of the artery, after the application of the ligature, is insisted on as absolutely necessary for the success of the operation. How can this be the case, if adhesion is the only process we are inducing? Next we have seen that the bare exposure of an artery, without the application of any ligature, is enough to induce its contraction. Are we not, then, authorised to conclude that the less injury we do to an artery, provided we excite the stimulus for contraction, the better is the vessel enabled to complete this salutary process? And is it not at least probable that by the continuance of the ligature for six hours, the injury may be such as to incapacitate the artery from a process which must require a degree of power scarcely to be expected after so much violence?

We therefore consider the experiment as unfairly conducted, though with the best intentions, and are even ready to admit that Dr. Jones, by his premature analogical reasoning, may have given rise to this error. Again we repeat it, let us le[illegible] what the powers of the arteries are in health and under disease, and not impute actions to causes altogether unsatisfactory, if not insufficient. If the reader has taken the trouble to examine our remarks on Dr. Jones's book, he may comprehend us still better.

There are several other passages in the history of this case which are well worth notice. On the first examination of the diseased limb, it was remarked that "the pulsations were strong, with considerable pain in the surrounding parts, extending to the limb beneath; and the circulation in the saphena vein seemed sluggish." This confirms an observation to be met with in the case of spurious aneurism cured by pressure above the injury, the relater of which suspects that there is a kind of sympathy between arteries and their corresponding veins.* In another passage Mr. H. asks,

"May we not infer from this circumstance, in opposition to the opinion of modern physiologists, that there is a contractile power inherent in the coats of arteries independent of their elastic one?"†

---

* See London Medical Journal, vol. vi. p. 541.

"† Dr. Jones certainly leaned to this latter opinion, as may be seen by a reference to the Preface of his book, page 4."

This

This contractile power is *proved* by Mr. Hunter in more experiments than it would be convenient even to count.*

The last remark we shall make is on the different result of operations on man or on brutes. The various causes of this need hardly be explained. In proportion as the machine is the more complicated, every new operation must be more uncertain: and, when the action of all the restorative powers is required, there cannot be a doubt that despondence and terror must considerably "counteract the calm and uniform progress of nature."

We shall here close our remarks on securing arteries for the present. It has already led us so far, that we can only notice the introductory paragraph to the chapter on necrosis.

"As the accompanying plate (says Mr. H.) represents the new osseous shell and sequestra of the most complete and interesting case of necrosis, that, I believe, is to be met with on record: I have thought that a brief statement of its leading points, with a few remarks on the formation of new bone in this disease, might not be deemed unworthy of consideration, the more especially so, as it is a subject upon which much contrariety of opinion has existed."

On reading this as the "best case on record," and on comparing "the plate" with the explanation, it was impossible not to recollect the late Mr. Hunter's lectures on the various diseases of the bones, or the *number* of *specimens* he exhibited in illustration of his doctrine. We shall, however, conclude with a most sincere wish—*Oh! si tantum ipsum audisses.*

---

*An Essay on the Shaking Palsy;* by JAMES PARKINSON, Member of the Royal College of Surgeons. 8vo. pp. 66. Sherwood and Co. 1817.

THE following apology of the author, contained in his preface, is transcribed on his account, and as a reason for our early notice of the tract.

"The advantages which have been derived from the caution with which hypothetical statements are admitted, are in no instance more obvious than in those sciences which more particularly belong to the healing art. It, therefore, is necessary, that some conciliatory explanation should be offered for the present publication: in which, it is acknowledged, that mere conjecture takes the place of experiment; and, that analogy is the substitute for anatomical examination, the only sure foundation for pathological knowledge.

"When, however, the nature of the subject, and the circumstances under

* See Treatise on the Blood, &c. part i. passim.

under which it has been here taken up, are considered, it is hoped that the offering of the following pages to the attention of the medical public, will not be severely censured. The disease, respecting which the present enquiry is made, is of a nature highly afflictive. Notwithstanding which, it has not yet obtained a place in the classification of nosologists; some have regarded its characteristic symptoms as distinct and different diseases, and others have given its name to diseases differing essentially from it; whilst the unhappy sufferer has considered it as an evil, from the domination of which he had no prospect of escape.

"The disease is of long duration: to connect, therefore, the symptoms which occur in its later stages with those which mark its commencement, requires a continuance of observation of the same case, or at least a correct history of its symptoms, even for several years. Of both these advantages the writer has had the opportunities of availing himself; and has hence been led particularly to observe several other cases in which the disease existed in different stages of its progress. By these repeated observations, he hoped that he had been led to a probable conjecture as to the nature of the malady, and that analogy had suggested such means as might be productive of relief, and perhaps even of cure, if employed before the disease had been too long established. He, therefore, considered it to be a duty to submit his opinions to the examination of others, even in their present state of immaturity and imperfection."

After this, the reader will consider himself as perusing a few hints which the author is anxious to communicate, in order to learn the opinions of others, and particularly that such practitioners as have an opportunity of inspecting the body of a person dying under the disease may not fail to communicate the result of his observations.

We shall omit all that is said concerning nosological definition, and at once present our readers with the history of the disease and a case.

"So slight and nearly imperceptible are the first inroads of this malady, and so extremely slow is its progress, that it rarely happens, that the patient can form any recollection of the precise period of its commencement. The first symptoms perceived are, a slight sense of weakness, with a proneness to trembling in some particular part; sometimes in the head, but most commonly in one of the hands and arms. These symptoms gradually increase in the part first affected; and, at an uncertain period, but seldom in less than twelvemonths or more, the morbid influence is felt in some other part. Thus, assuming one of the hands and arms to be first attacked, the other, at this period, becomes similarly affected. After a few more months the patient is found to be less strict than usual in preserving an upright posture: this being most observable whilst walking, but sometimes whilst sitting or standing. Sometime after theappearance of this symptom, and during its slow increase, one of the legs is discovered slightly to tremble, and is also found to

4 suffer suffer fatigue sooner than the leg of the other side: and, in a few months, this limb becomes agitated by similar tremblings, and suffers a similar loss of power.

"Hitherto, the patient will have experienced but little inconvenience; and, befriended by the strong influence of habitual endurance, would, perhaps, seldom think of his being the subject of disease, except when reminded of it by the unsteadiness of his hand, whilst writing or employing himself in any nicer kind of manipulation. But as the disease proceeds, similar employments are accomplished with considerable difficulty, the hand failing to answer with exactness to the dictates of the will. Walking becomes a task which cannot be performed without considerable attention. The legs are not raised to that height, or with that promptitude which the will directs, so that the utmost care is necessary to prevent frequent falls.

"At this period, the patient experiences much inconvenience, which unhappily is found daily to increase. The submission of the limbs to the directions of the will can hardly ever be obtained in the performance of the most ordinary offices of life. The fingers cannot be disposed of in the proposed directions, and applied with certainty to any proposed point. As time and the disease proceed, difficulties increase: writing can now be hardly at all accomplished; and reading, from the tremulous motion, is accomplished with some difficulty. Whilst at meals the fork, not being duly directed, frequently fails to raise the morsel from the plate: which, when seized, is with much difficulty conveyed to the mouth. At this period the patient seldom experiences a suspension of the agitation of his limbs. Commencing, for instance, in one arm, the wearisome agitation is borne until beyond sufferance, when, by suddenly changing the posture, it is for a time stopped in that limb, to commence, generally, in less than a minute in one of the legs, or in the arm of the other side. Harrassed by this tormenting round, the patient has recourse to walking, a mode of exercise to which the sufferers from this malady are in general partial; owing to their attention being thereby somewhat diverted from their unpleasant feelings, by the care and exertion required to ensure its safe performance.

"But, as the malady proceeds, even this temporary mitigation of suffering from the agitation of the limbs is denied. The propensity to lean forward becomes invincible, and the patient is thereby forced to step on the toes and fore part of the feet, whilst the upper part of the body is thrown so far forward as to render it difficult to avoid falling on the face. In some cases, when this state of the malady is attained, the patient can no longer exercise himself by walking in his usual manner, but is thrown on the toes and fore part of the feet; being, at the same time, irresistibly impelled to take much quicker and shorter steps, and thereby to adopt unwillingly a running pace. In some cases it is found necessary entirely to substitute running for walking; since otherwise the patient, on proceeding only a very few paces, would inevitably fall.

"In this stage, the sleep becomes much disturbed. The tremulous motion of the limbs occur during sleep, and augment until they

 awaken awaken the patient, and frequently with much agitation and alarm. The power of conveying the food to the mouth is at length so much impeded that he is obliged to consent to be fed by others. The bowels, which had been all along torpid, now, in most cases, demand stimulating medicines of very considerable power: the expulsion of the fæces from the rectum sometimes requiring mechanical aid. As the disease proceeds towards its last stage, the trunk is almost permanently bowed, the muscular power is more decidedly diminished, and the tremulous agitation becomes violent. The patient walks now with great difficulty, and, unable any longer to support himself with his stick, he dares not venture on this exercise, unless assisted by an attendant, who, walking backwards before him, prevents his falling forwards, by the pressure of his hands against the fore part of his shoulders. His words are now scarcely intelligible; and he is not only no longer able to feed himself, but, when the food is conveyed to the mouth, so much are the actions of the muscles of the tongue, pharynx, &c. impeded by impaired action and perpetual agitation, that the food is with difficulty retained in the mouth until masticated; and then as difficultly swallowed. Now also, from the same cause, another very unpleasant circumstance occurs: the saliva fails of being directed to the back part of the fauces, and hence is continually draining from the mouth, mixed with the particles of food which he is no longer able to clear from the inside of the mouth.

"As the debility increases and the influence of the will over the muscles fades away, the tremulous agitation becomes more vehement. It now seldom leaves him for a moment; but even when exhausted, Nature seizes a small portion of sleep, the motion becomes so violent as not only to shake the bed-hangings, but even the floor and sashes of the room. The chin is now almost immoveably bent down upon the sternum. The slops with which he is attempted to be fed, with the saliva, are continually trickling from the mouth. The power of articulation is lost. The urine and fæces are passed involuntarily; and, at the last, constant sleepiness, with slight delirium, and other marks of extreme exhaustion, announced the wished-for release.

"Case i.—Almost every circumstance noted in the preceding description, was observed in a case which occurred several years back, and which, from the particular symptoms which manifested themselves in its progress; from the little knowledge of its nature, acknowledged to be possessed by the physician who attended; and, from the mode of its termination; excited an eager wish to acquire some further knowledge of its nature and cause.

"The subject of this case was a man rather more than fifty years of age, who had industriously followed the business of a gardener, leading a life of remarkable temperance and sobriety. The commencement of the malady was first manifested by a slight trembling of the left hand and arm, a circumstance which he was disposed to attribute to his having been engaged for several days in a kind of employment requiring considerable exertion of that limb. Although repeatedly repeatedly questioned, he could recollect no other circumstance which he could consider as having been likely to have occasioned his malady. He had not suffered much from rheumatism, or been subject to pains of the head, or had ever experienced any sudden seizure which could be referred to apoplexy or hemiplegia. In this case, every circumstance occurred which has been mentioned in the preceding history."

A chapter follows on "Pathognomic Symptoms." Another on the distinguishing characters: a third, on the proximate and remote causes; and a concluding one, on the means of cure.

The author considers the causes to be principally in some morbid change in the medulla spinalis, its membranes, or the containing theca; urging, with much propriety, that, however ready we may be to acknowledge ourselves uninformed on these points, we should rather impute such ignorance to a want of inquiry than to an impossibility of arriving at the true source. When the latter is discovered, the disease will be readily cured, or we shall learn the cause of our want of success. When the disease can be traced in any member, so as to give a fair implication of the part of the spine likely to be affected, Mr. P. proposes bleeding in the neighbourhood of the latter, and the application of blisters or issues. In the first of these we perfectly agree, and are sure the candid author will find much gratification in learning, that bleeding with cupping glasses on each side of the spine has been a practice with us in many cases similar. At this time, two females are receiving relief at a public institution, one who has never menstruated, though arrived at the age for that process; the other, in whom menstruation has been apparently impeded by a laborious employment and scanty diet. Each has pains in the vertebræ, and one of them an incapacity to preserve her posture erect, or to walk without difficulty. The other has numbness in the lower limbs, with trembling and involuntary motion. It should be added, that in the latter there is a considerable bony projection in one part of the spine, though the whole figure is tolerably erect. Both are considerably relieved by topical bleeding.

It is with reluctance that we can allow such scanty limits to so many valuable hints as we have met with in this pamphlet. Its confined size, however, will bring it within the reach of every practitioner, and we heartily recommend it to universal perusal.

